# Identification and functional analysis of a new putative caveolin-3 variant found in a patient with sudden unexplained death

**DOI:** 10.1186/1423-0127-21-58

**Published:** 2014-06-10

**Authors:** Vincenzo Lariccia, Annamaria Assunta Nasti, Federica Alessandrini, Mauro Pesaresi, Santo Gratteri, Adriano Tagliabracci, Salvatore Amoroso

**Affiliations:** 1 Department of Biomedical Sciences and Public Health, School of Medicine, University “Politecnica delle Marche”, Ancona, Italy; 2 Department of Health Sciences, University “Magna Graecia”, Catanzaro, Italy

**Keywords:** Caveolin-3, Sudden cardiac death, ERKs

## Abstract

**Background:**

Sudden cardiac death (SCD) is the clinical outcome of a lethal arrhythmia that can develop on the background of unrecognized channelopathies or cardiomyopathies. Several susceptibility genes have been identified for the congenital forms of these cardiac diseases, including caveolin-3 (Cav-3) gene. In the heart Cav-3 is the main component of caveolae, plasma membrane domains that regulate multiple cellular processes highly relevant for cardiac excitability, such as trafficking, calcium homeostasis, signal transduction and cellular response to injury. Here we characterized a new putative Cav-3 variant, Cav-3 V82I, found in a patient with SCD.

**Results:**

In heterologous systems Cav-3 V82I was expressed at significantly higher level than Cav-3 WT and accumulated within the cells. Cells expressing Cav-3 V82I exhibited a decreased activation of extracellular-signal-regulated kinases (ERKs) and were more vulnerable to sub-lethal osmotic stress.

**Conclusion:**

Considering that abnormal loss of myocytes can play a mechanistic role in lethal cardiac diseases, we suggest that the detrimental effect of Cav-3 V82I variant on cell viability may participate in determining the susceptibility to cardiac death.

## Background

The most challenging type of SCD occurs when it cannot be traced to any evident cause other than a lethal arrhythmia, which may represent the sole sentinel event of an otherwise silent disease [1,2]. Congenital cardiac channelopathies and cardiomyopathies are known to play significant roles in SCD [2-4]. In the first cases genotype-phenotype correlation studies have identified most of the known disease-causing defects in genes encoding cardiac ion channels or other membrane components [3], while in the second cases cardiac structural abnormalities have been detected in subjects carrying mutations in sarcomeric proteins [4]. However, the identified variants commonly have reduced penetrance and are associated with heterogeneous clinical manifestations [2]. Such variability in disease expression suggests that other modifying and triggering causes must be considered [2]. For instance, genetic predisposition and environmental factors may negatively influence the arrhythmic risk by increasing heart susceptibility to stressing conditions [5-8]. Aberrant cell death can potentially provoke at least three changes in the heart that are relevant to lethal arrhythmic processes, namely cardiac contractility compromise, conduction disturbance and cardiac remodeling [9-11].

Recent studies have discovered mutations in *CAV3* gene encoding caveolin-3 (Cav-3) in subject affected by hereditary arrhythmias - such as long QT syndrome (LQTS) and sudden infant death syndrome (SIDS) [12,13] - or congenital cardiomyopathies [14,15]. Cardiac manifestations are not commonly observed among individuals affected by caveolinopathies, which often display abnormal skeletal muscle phenotypes [15-19].

Cav-3 is predominantly expressed in cells experiencing cyclic mechanical stress (such as striated muscle myocytes) and is one of the three major isoforms of caveolins [20]. These proteins localize within caveolae, plasma membrane microdomains considered as key platforms for numerous cellular processes such as endocytosis, lipid metabolism, mechanosensing and survival response to stressful stimuli [20-27]. Caveolar membranes contain and regulate different signalling enzymes, including ERKs (Extracellular-signal-Regualted Kinases, also known as p44/42 MAP kinases) [28]. Caveolins bind to and negatively regulate ERK activity [28,29]. ERKs can be considered as master regulatory kinases, critically involved in cell fate determination processes in response to various stressful stimuli [30]. In particular, alterations in ERK signalling negatively impact on cell viability under stressed conditions such as hyperosmotic shock [30,31].

In the present study, we investigated whether a new putative Cav-3 variant, Cav-3 V82I, found in a patient with SCD in adulthood, renders cells more vulnerable to osmotic stress. In particular, we collected evidence that this variant accumulates within the cell, impairs ERK activation and increases cell death susceptibility to sub-lethal osmotic stress.

## Methods

### Analysis of sequence

Genomic DNA was extracted by phenol/chloroform from peripheral blood, obtained after informed consent from 50 unrelated patients with suspected or diagnosed LQTS, for which a genetic screening was requested by cardiologists for definitive diagnosis. No mutations were found in the entire coding regions of the major LQTS associated genes (KCNQ1, KCNH2, SCN5A, KCNE1, KCNE2, and KCNJ2) and in ANK2 and RyR2 genes [3,4]. DNA was submitted to open reading frame/splice site mutational analysis on CAV3 gene by PCR and direct DNA sequencing, using coding region flanking primers (Table 1). PCR was performed in a final volume of 25 μl containing 1X Buffer, 1.5 mM MgCl_2_, 1 μM each primer, 0.2 mM each dNTP, 2.5 U Taq polymerase (all from Euroclone, Milan, Italy) and 10 ng of DNA template. PCR was performed on a 9700 thermal cycler (Applied Biosystems, Monza, Italy) and involved 1 cycle at 95°C for 5 min, followed by 35 denaturation cycles at 94°C for 30 sec, annealing at 57°C for 30 sec, extension at 72°C for 30 sec and a final extension at 72°C for 10 min. Primers for sequencing reaction were the same of PCR. Sequencing reaction was performed using BigDye terminators ready reaction kit (Applied Biosystems, Monza, Italy) according to manufacturer’s protocol. Sequencing products were submitted to CE on Applied Biosystems 3130 Genetic Analyzer. Sequences were aligned and compared with the Cav-3 reference sequence NM_033337 in GenBank by Seqscape software v2.5.0 (Applied Biosystems, Monza, Italy).

**Table 1:** **Primers used for *CAV3 *** **PCR and sequencing reaction**

**Primer**	**Sequenza 5′ > 3′**
Cav3-1 F	GCCTATTTAGCTGGCAGGGAC
Cav3-1R	CCACGTCTCGCAAACCTGAC
Cav3_2.1_	CCTGACCTCTAGGGGATTCTG
Cav3_2.1_	CCCCACCTGGCTTTAGACC
Cav3_2.2 F	CCTTCTGCAACCCACTCTTC
Cav3_2.2R	GTGGAGAGGTTGGCCCC

All experiments were carried out in strict accordance with the Clinical Practice Guidelines of A.O.U. “Ospedali Riuniti di Ancona”, and with the Oviedo Convention on Human Rights and Biomedicine, ratified by Italian law 145 of 28 March 2001.

### Site-direct mutagenesis

Plasmid containing Cav-3 wild-type cDNA was purchased from Origene. In vitro site-direct mutagenesis was performed by QuickChange Site-Direct Mutagenesis kit (Agilent Technologies, Milan, Italy) following manufacturer’s instructions. The presence of the G244A mutation (V82I) was confirmed by sequence analysis.

### Cell culture

BHK cells were cultured in a humified 5% CO_2_ atmosphere in DMEM supplemented with 10% fetal bovine serum, 6 mM glutamine, 1 mM pyruvate, 100 U/ml penicillin and 100 μg/ml streptomycin. For immunofluorescence studies, cells were plated on glass coverslips 16 h before transfection.

### Transient transfection and western blot

The plasmids containing respectively the Cav-3 WT and Cav-3 V82I were transfected to BHK cells with Attractene (Qiagen, Milan, Italy) following manufacturer’s instructions. Transfection efficiency, quantified by co-transfecting Cav-3 and EGFP expressing plasmids, was ~70-80% both for the WT and mutant. Twenty-four hours post transfection, whole cell lysates for western blot analysis were obtained using a cell lysis solution containing (in mM): NaCl, 150; Tris–HCl (pH 7.4), 10; EDTA (pH 8.0), 1; SDS 1%, and a protease inhibitor cocktail mixture. All samples were prepared in buffer containing SDS and 2-mercaptoethanol and boiled at 100°C for 10 min prior to loading. Immunoblotting on 8% polyacrylamide was performed as described previously [32] and the following primary antibodies were used: mouse anti-CAV3 (1:500), purchased from Santa Cruz Biotechnology (Santa Cruz, California, USA) and mouse anti-tubulin (1:10000; Sigma). The band images were digitally captured and band intensities were quantified using a ChemiDoc station and the Quantity one analysis software. The relative amount of caveolin-3 protein was normalized on tubulin. In some experiments, 10 μg/ml cycloheximide (stock solution 100 mg/ml in DMSO; Millipore, Billerica, MA, USA) was added directly to BHK cells 24 h after transfection with Cav-3 WT or Cav-3 V82I and the relative amount of caveolin-3 protein was determined by collecting cell lysates at the indicated time points [33,34].

### Caveolin-3 Triton solubility

Caveolin-3 partitions into Triton-soluble fraction were determined 24 h after transfection with the indicated plasmid as described before [14,35,36]. Briefly, transfected BHK cells were washed twice with ice-cold PBS and lysed for 30 min on ice in 1% Triton X-100 buffer (pH 6.5) containing 150 mM NaCl, 25 mM MES, protease inhibitors. Samples were then centrifuged at 14,000 × g for 10 min at 4°C and pellet (the insoluble fraction; I) and supernatant (soluble fraction; S) were resolved and analyzed by caveolin-3 immunoblotting as described before.

### Immunofluorescence microscopy

24 hours after transfection, BHK cells plated on glass coverslips were washed with PBS and fixed for 15 min with ice-cold PBS 4% paraformaldehyde, 2% sucrose. Cells were then incubated with PBS 3% BSA and 0.1% Triton for 30 min at RT and following incubated over night with the mouse antibody anti-Cav-3 (Santa Cruz Biotechnology) 1:500 in PBS 3% BSA. Finally, cells were incubated for 1 h in the dark with the Alexa Fluor 488 anti-mouse IgG (Invitrogen, Monza, Italy) 1:500 in PBS 3% BSA and then analyzed on a Zeiss LSM510 META confocal microscope (Zeiss, Arese, MI, Italy). For Nile red staining, the dye was added directly to fixed cells (1:100 dilution in PBS from a 1 mg/ml stock solution in acetone) after Cav-3 immunolabelling, and samples were incubated in the dark for 10 min at room temperature.

### Determination of mitochondrial activity and cell death

Mitochondrial activity was quantified by measuring dehydrogenase activity retained in the cultured cells, using the MTT assay [37]. The assay is based on the ability of living cells to convert dissolved MTT into insoluble formazan. Briefly, 24 h after transfection, BHK cells were washed with PBS and treated for 30 min in the cell culture incubator with 1 ml culture medium or mannitol solution (1.1 M in culture medium). After an additional wash with PBS, cells were then incubated with 1 ml of MTT (3-(4,5-Dimethylthiazol-2-yl)-2,5-diphenyltetrazolium bromide) solution (0.5 mg/ml in PBS). After 1 h incubation at 37°C, cells were finally washed with PBS and the formazan produced was dissolved in 1 ml of dimethylsulfoxide (DMSO). The absorbance was read at 540 nm. Data were expressed as the percentage of cell injury to sham-treated cultures.

To evaluate cell death under basal and mannitol stress conditions, cells were treated as described above and then double stained with 36 μM Fluorescein diacetate and 7 μM Propidium iodide for 20 min at 37°C in PBS. Stained cells were examined immediately with a standard inverse fluorescence microscope and analyzed as described before [38].

### Determination of ERK activity

ERK activity was evaluated by *in vitro* phosphorylation of Elk-1 protein (an ERK substrate) in cell lysates using a commercially available nonradioactive assay kit (Cell Signaling, Boston, MA, USA). Briefly, 1 × 10^6^ BHK cells were plated into 100 mm dishes 16 h before transfection with EGFP, Cav-3 WT or Cav-3 V82I expressing vectors. Transfected cells were exposed to standard or mannitol-containing culture media as previously described. pERK affinity precipitation was performed incubating cell extracts (overnight at 4°C) with sepharose bead conjugate phospho-p44/42 MAP Kinase monoclonal antibody. The immunoprecipitates were then used in an in vitro kinase assay using Elk-1 as substrate. pERK activity was finally evaluated quantifying by immunoblotting the level of Elk-1 phosphorylation with anti-phospho-Elk-1 (pElk) mouse antibody.

Since the doubly phosphorylated forms of ERK represent the active enzymes, we also used anti-phospho-ERK immunoblotting as direct ERK activation marker. To detect the active form of ERKs, cell lysates were subjected to western blot analysis using pERK and ERK antibodies (Santa Cruz, California, USA). The amount of ERK phosphorylation was quantified by densitometry and normalized by expressing the data as a ratio of pERK over the total ERK.

### Statistical analysis

Data were analysed by t-test for paired or unpaired data as appropriate. For multiple group comparisons, two way ANOVA followed Dunnett post-hoc test was used as appropriate.

### Chemicals

All the other chemicals were of analytical grade and were purchased from Sigma, Italy.

## Results

### Identification of a novel CAV3 mutation

**Figure 1:**
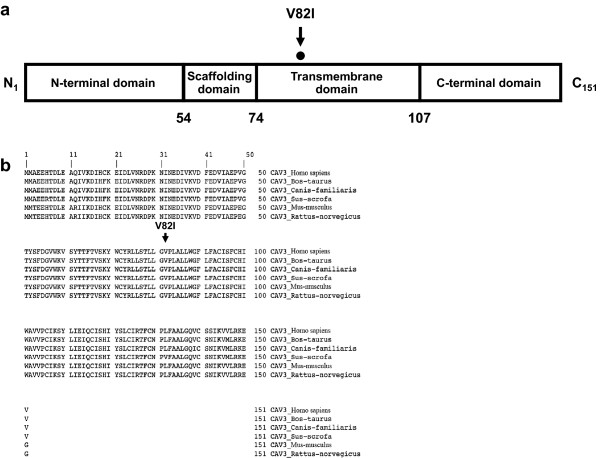
**Caveolin-3 topological domains and localization of the V82I variation identified in LQTS patient. (a)** Schematic topological organization of the caveolin-3 protein. Numbers above the line indicate amino acid residues in caveolin-3 protein sequence that define four domains (23): N-terminal (aa 1–53), scaffolding (aa 54–73), transmembrane (aa 74–106) and C-terminal (aa 107–151). V82I mutation affects a residue within the transmembrane domain. **(b)** Sequence alignment of caveolin-3 protein of the indicated vertebrates is shown. V82I mutation modifies a conserved amino acids in caveolin-3.

Sequencing analysis of CAV3 gene in 50 probands with suspected or diagnosed LQTS identified 9 single nucleotide variations (SNVs), 3 on the exon 1 amplicon and 6 on the exon 2 amplicon. Five SNVs were localized in the flanking region of exon 1 and exon 2 and were not considered in this study. The remaining four SNVs (C27T corresponding to SNP rs1974763, C99T to rs1008642, T123C to rs13087941 and G244T to rs112626848) were found in the coding region of the CAV3 gene; three of them (rs1974763, rs1008642 and rs13087941) were synonymous mutations with no pathogenic implications; instead rs112626848 was heterozygous missense mutation at nucleotide 244 (G > A) (Additional file 1) leading to amino acid change, valine to isoleucine, at codon 82 (V82I), involving a residue conserved across several species in the transmembrane domain (Figure 1 and [12]).

### Expression and distribution of wild type and V82I mutant of Caveolin-3

**Figure 2:**
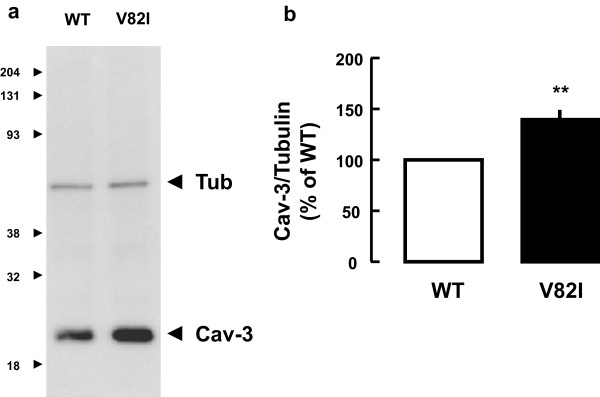
**Recombinant expression of Cav-3 WT and Cav-3 V82I. (a)** BHK cells were transiently transfected with either Cav-3 WT or Cav-3 V82I expressing plasmids. Twenty-four hours after transfection, cells were lysed and subjected to western blot analysis using anti-caveolin-3 antibody. The mutant form was expressed at significantly higher level than achieved with the wild-type caveolin-3. **(b)** Quantization revealed that Cav-3 V82I was expressed at about 40% the expression level observed for Cav-3 WT. Data are representative of 15 independent experiments (*** P < 0.001).

**Figure 3:**
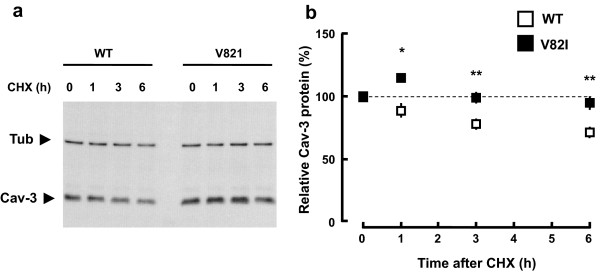
**Stability of Cav-3 WT and Cav-3 V82I.** Transfected BHK cells were treated with cycloheximide (10 μg/ml; CHX) for the indicated length of time. Cells were then collected in same volumes of lysis buffer and equal volumes of extracts were analyzed by immunoblotting. Representative blot is shown in **(a)**. Levels of residual caveolin-3 at the indicated time points (% of time 0) for Cav-3 WT and Cav-3 V82I are shown in **(b)**. Data are representative of four independent experiments. *, P < 0.05 vs WT at the respective time point; **, P < 0.01 vs WT at the respective time points.

To investigate the impact of V82I mutation on Cav-3 protein level and cellular localization, we introduced the V82I substitution detected in the patient into the human Cav-3 WT sequence. We next transfected BHK cells with Cav-3 WT or Cav-3 V82I and compared levels of wild-type and mutant in SDS lysates, which represent the total caveolin-3 pool. As shown in Figure 2, Cav-3 V82I mutant was expressed at significantly higher level (about 1.4 fold) than Cav-3 WT. The increased expression in caveolin-3 protein caused by V82I mutation is somehow a new finding since most of caveolin-3 mutations often cause a severe loss of Cav-3 protein [19]. To examine whether differences in protein stability between WT or V82I mutant accounted for the higher expression level of Cav-3 V82I, we performed cycloheximide (CHX) block experiments [33,34]. WT or V82I transfected BHK cells were treated with CHX for 1–6 h to inhibit protein synthesis, harvested at different time points, and resulting lysates were probed with anti-Cav-3 antibody to monitor caveolin-3 levels, or anti-tubulin antibody as a loading control. As shown in Figure 3, during CHX chase, Cav-3 WT protein declined over time, decreasing to 70% levels by 6 h, while Cav-3 V82I levels were significantly more stable over the 6 h time course. These results suggest that V82I mutation confers increased stability to caveolin-3 and are consistent with the higher protein expression level observed with Cav-3 V82I as compared to Cav-3 WT. It is interesting to note that 1 h of CHX treatment significantly increased the expression of the V82I mutant (but not of the WT form): the reason of such increase is currently unknown.

**Figure 4:**
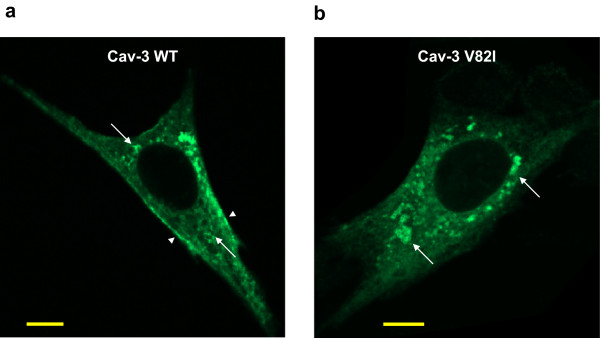
**Immonolocalization of Cav-3 WT and Cav-3 V82I.** BHK cells were transiently transfected with Cav-3 WT **(a)** or Cav-3 V82I **(b)** and immunostained with the antibodies against caveolin-3 followed by Alexa Fluor 488 conjugated secondary antibodies. Cav-3 V82I mutant was retained intracellularly and not properly targeted to the plasma membrane as Cav-3 WT (arrowheads). Vesicular-like structures (LB) stained with the caveolin-3 antibodies were also observed, especially for Cav-3 V82I mutant (arrows); Scale bar: 10 μm. Four independent transfected cultures were analyzed.

**Figure 5:**
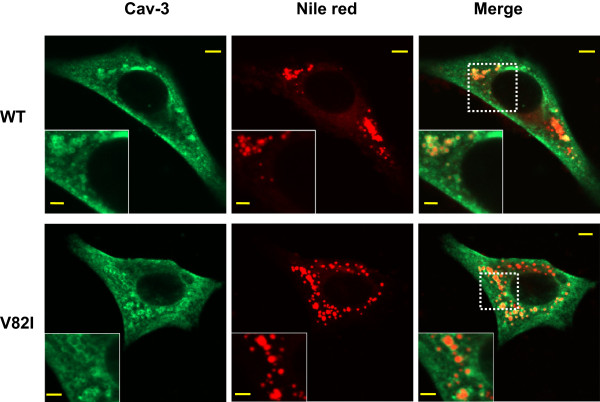
**Colocalization analysis of Cav-3 WT and Cav-3 V82I with the LB marker Nile red.** Cav-3 transfected cells were labeled with Cav-3 antibodies followed by Alexa Fluor 488 conjugated secondary antibodies. LBs were then stained with Nile red (1:100 dilution in PBS from 1 mg/ml stock solution in acetone). BHK cells expressing Cav-3 V82I (bottom) showed a marked localization in LB structures (bottom) as compared to Cav-3 WT (top). Scale bar: 5 μm (2.5 μm in the inserts). Two independent transfected cultures were analyzed.

We further investigated the sub-cellular distribution of wild type and mutant V82I caveolin-3 by means of confocal microscopic analysis. In BHK cells, the wild type caveolin-3 was localized both at the plasma membrane and within the cells, while Cav-3 V82I was mainly retained within the cell (Figure 4; see Additional file 2 for complete Z-stack image series). In most cells, a ring-like labelling was also observed, especially for Cav-3 V82I mutant (Figure 4). These intracellular structures stained with the Cav-3 antibody varied in size and were often clustered together. To determine whether these spherical structures were the so-called lipid bodies (LB), organelles targeted by caveolins (both wild types and mutant forms) [34,39,40], we carried out double-labelling analysis with antibodies to caveolin-3 proteins and with Nile red, a marker for LB [34,39,40]. As reported in Figure 5, both in Cav-3 WT and in Cav-3 V82I expressing cells, the intracellular Cav-3 pools highly localized on the surface of Nile red stained vesicles. However, we found that not all the Cav-3 positive structures were also positive for Nile red, as well as that some LB were stained only with Nile red. Similar results were obtained in a rat cardiomyoblast cell line, H9c2 cells (Additional file 3), suggesting that the observed differences in protein expression between Cav-3 V82I and WT forms were not related to the cell type used.

**Figure 6:**
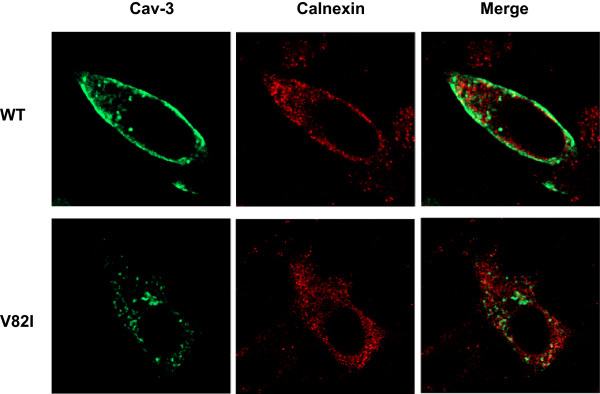
**Colocalization analysis of Cav-3 WT and Cav-3 V82I with the ER marker calnexin.** BHK cells were transiently transfected with Cav-3 WT (top) or Cav-3 V82I (bottom) and subjected to double labelling with antibodies raised against caveolin-3 (green) and calnexin (red). As shown in the merged images, no significant colocalization with calnexin was observed for both wilt-type and mutated caveolin-3 proteins. Data are representative of three independent cultures.

It has been reported that unstable Cav-3 mutants, usually characterized by a marked reduction in their expression and retained in the Golgi complex [15,19,35,36], are expressed at higher levels and accumulate within the endoplasmic reticulum (ER) after treatment with proteasome inhibitors [41]. Considering that the V82I mutation increased Cav-3 expression and that LB have an ER origin [39], we sought to investigate whether the distribution of Cav-3 V82I protein was also consistent with the retention of the protein in the ER. To address this hypothesis, transiently transfected BHK cells were subjected to double labeling with antibodies directed against caveolin-3 and calnexin, an ER specific marker protein [42]. Despite the increased expression and intracellular caveolin-3 retention observed in Cav-3 V82I transfected cells, V82I mutant was not distributed to ER since no significant colocalization was observed between V82I and calnexin (Figure 6). In cells transfected with the WT form, caveolin-3 was mostly expressed at cell surface and did not colocalize with calnexin (Figure 6), in line with previous results [41].

**Figure 7:**
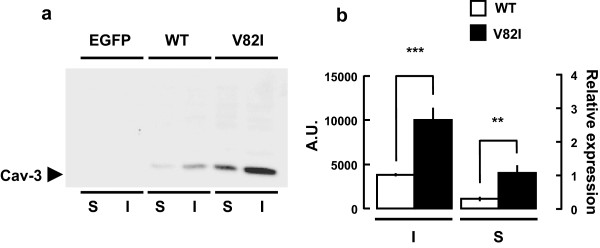
**Cav-3 V82I partitions into Triton-soluble (S) and Triton-insoluble (I) cell fractions.** Twenty-four hours after transfection, the Triton-soluble and insoluble fractions were collected from Cav-3 WT and Cav-3 V82I transfected BHK cells as described in Materials and Methods. Equal volumes of the soluble and insoluble fractions were analyzed by immunoblotting. Representative blot is shown in **(a)**. Cav-3 V82I was significantly more abundant than the WT both in the S and in the I fractions **(b)**. Data are representative of five independent experiments. **, P < 0.01 V82I vs WT in the soluble fraction; ***, P < 0.001 V82I vs WT in the insoluble fraction.

We next characterized the biochemical properties of the Cav-3 V82I assessing its detergent solubility pattern. Caveolins mainly reside within caveolar lipid rafts which are insoluble in non-ionic detergents such as Triton X-100, while several caveolin-3 mutants with decreased surface expression usually display an increased Triton X-100 solubility [14,35,36]. By western blot analysis we found that in Cav-3 WT transfected cells caveolin-3 protein was mainly distributed in the Triton-insoluble fraction (I) (Figure 7), consistent with data reported before [36,43]. For Cav-3 V82I, we observed a significant increased of caveolin-3 expression both in the Triton-insoluble and in the Triton-soluble (S) fractions (respectively 2.6 and 3.5 fold as compared to WT; Figure 7). The increased distribution of Cav-3 V82I in the Triton-soluble fraction (expressed as caveolin-3 soluble/pellet ratio) was not statistically different (42.4 ± 10.1% vs 29.1 ± 5.9%, V82I vs WT, respectively; n = 5, P = 0.29).

### Effect of Cav-3 V82I on ERK activity and cell viability

**Figure 8:**
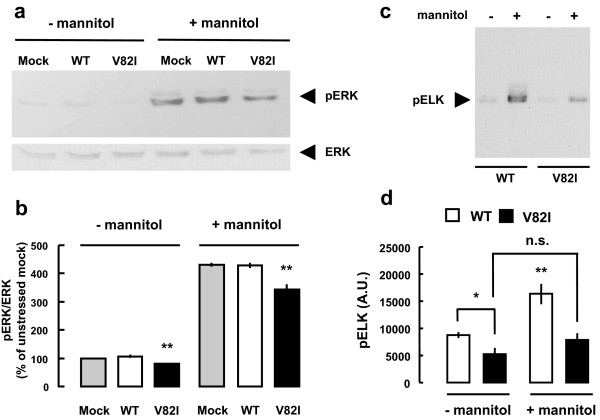
**Effects of Cav-3 WT or Cav-3 V82I expression on ERK1/2 activation.** BHK cells were transiently transfected with Cav-3 WT or Cav-3 V82I. **(a)** Cell lysates were subjected to SDS-PAGE and analyzed by immunoblotting using anti-ERK and anti-pERK antibodies. Expression of total cellular level of ERK remained unaltered after the transfection with either Cav-3 WT or Cav-3 V82I in all the conditions tested. On the contrary, a significant decrease in pERK (active form) was observed in the presence of the caveolin-3 mutant, both under resting conditions and after 30 min hyperosmotic stress with mannitol. **(b)** Quantization revealed that in Cav-3 V82I expressing cells there was a significant decrease of ERK activation as compared with Cav-3 WT and mock transfected cells. Data are representative of three independent experiments. **, P < 0.01 vs the respective mock and Cav-3 WT groups. Similar results were obtained in **(c,d)**. Cell lysates from control and mannitol treated groups were subjected to an in vitro ERK1/2 kinase assay as described in Materials and Methods. Representative blot is shown in **(c)** and relative band intensities of pElk analysis is shown in **(d)**. Data are representative of three independent cultures. *, P < 0.05 V82I vs WT in untreated cells; **, P < 0.01 WT vs all groups.

**Figure 9:**
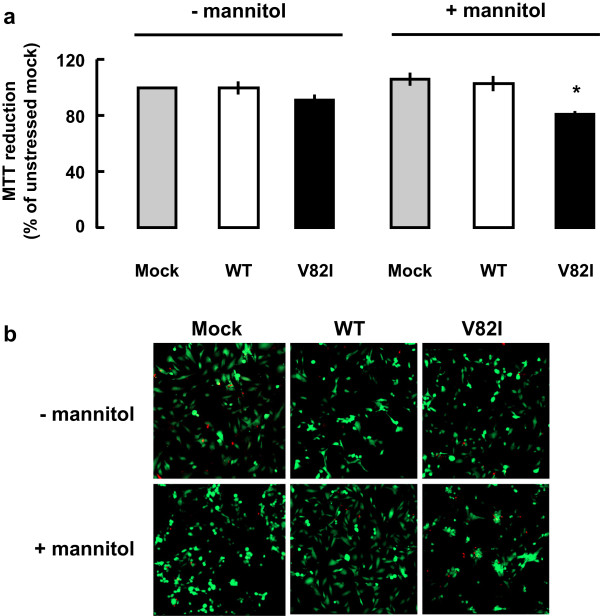
**Effects of Cav-3 WT or Cav-3 V82I expression on cells viability.** BHK cells were transiently transfected with Cav-3 WT or Cav-3 V82I and mitochondrial activity and cell death were evaluated by MTT and Propidie/Fluorosceine assays, respectively. **(a)** Expression of Cav-3 WT or Cav-3 V82I did not modified mitochondrial function under basal conditions. On the contrary, a significant decrease of mitochondrial activity was observed in Cav-3 V82I expressing cells subjected to hyperosmotic stress. Data are representative of five independent experimental sessions. *, P < 0.05. **(b)** Images show the intravital staining that yields green yellow fluorescence for vital cells and red fluorescence for dead cells under basal and hyperosmolar conditions. Images are representative of three independent experiments.

As previously reported, caveolin proteins negatively regulate ERK activity [28,29,44]. Considering that cell viability is compromised when ERK signalling pathways are disrupted [30,31], we first tested the effects of Cav-3 V82I on ERK activation. Lysates from BHK cells transfected, either with Cav-3 WT or Cav-3 V82I, were analyzed by western blot using specific monoclonal antibodies directed against total ERKs or their phosphorylated (active) form. Both Cav-3 WT and Cav-3 V82I did not modify total ERK expression. On the contrary V82I mutant significantly impaired both basal and mannitol-stimulated ERK activation as compared to Cav-3 WT (Figure 8). Similar results were obtained when we measured p44/42 MAP kinase activity using a nonradioactive assay kit that detects the specific phosphorylation of the p44/42 MAP kinase substrate Elk-1 (Figure 8). Interestingly, cells transfected with Cav-3 V82I were more vulnerable to the mannitol challenge used to activate ERK, since both mitochondrial activity (measured by MTT assay) and cell viability (measured by the Propidium/Fluoroscein assay) were significantly affected (Figure 9).

## Discussion

In this report, we identified and characterized a novel heterozygous mutation in caveolin-3 gene. This mutation (V82I) was found in an individual adult with suspected LQTS who suddenly died prior to completion of comprehensive cardiological examination. No mutations were found in the entire coding regions of the major LQTS associated genes (KCNQ1, KCNH2, SCN5A, KCNE1, KCNE2, and KCNJ2) and in ANK2 and RyR2 genes [2,3].

Valine-to-isoleucine substitution is considered as a conservative change in that the two amino acids differ by only a methylene group, and *a priori* it would not be expected to substantially alter the properties of the protein. However, despite their similarity, their side-chains differ in size, shape and hydrophobicity, and these differences account for the fact that valine and isoleucine are not always interchangeable in proteins [45]. Moreover, V82I mutation alters a conserved amino acid of the Cav-3 membrane-spanning segment (Figure 1 and [12]).

In humans, the majority of caveolinopathies often refers to a wide spectrum of skeletal muscle disorders [19,46] and only recently mutations in caveolin-3 were associated to cardiac pathologies including LQTS and SIDS [12,13] and cardiomyopayhies [14,15]. Several mechanisms have been proposed for the skeletal muscle degenerative processes, whereas the molecular bases for the cardiac phenotypes are largely unknown [14,19,46].

Biochemical analysis of transiently transfected cell line showed that Cav-3 V82I is expressed at higher level than Cav-3 WT (Figure 2 and Additional file 3) probably as consequence of higher protein stability as revealed by CHX assay (Figure 3). Moreover, V82I mutant did not alter the detergent solubility of caveolin-3 protein, and a significant increase of caveolin-3 expression was found both in the Triton-insoluble (I) and in the Triton-soluble (S) fractions (Figure 7). These results are in strike contrast to the majority of the known caveolin-3 mutants which cause 1- severe loss of protein, 2- decrease of protein half-life, 3- increased expression in the Triton-soluble fraction [14,19,35,36,41].

To study the functional alterations of Cav-3 due to V82I mutation, we examined the intracellular distribution of the mutant protein by confocal microscopy. Immunofluorescence studies revealed that Cav-3 V82I tends to accumulate within the cells, suggesting that the V82I mutation affects a critical residue required for correct cell surface expression as observed with caveolin-3 WT (Figures 4, 5, 6 and Additional files 2, 3). It is interesting to note that the threonine 78, a residue very close to the valine 82, is also critical for proper targeting of caveolin-3 protein to the plasma membrane, since Cav-3 T78K (but not Cav-3 T78M) mutant fails to distribute to cellular surface [15]. The retention in intracellular compartments is a common phenotype observed with different pathogenetic caveolin-3 mutants that often form aggregates within Golgi complex [15,19,35,36,41,47]. In contrast to the WT form, Cav-3 V82I showed a particular cellular distribution, characterized by reduced targeting to the plasma membrane and, in most transfected cells, by increased staining of ring-like LB structures (Figures 4, 5 and Additional files 2, 3). A very similar labelling pattern was reported for Cav-3^DGV^, an amino-terminal truncation mutant of caveolin-3, which fails to reach plasma membrane and highly localizes into LB [40,47]. Since Cav-3 V82I mutant is not properly distributed to cell surface, we suggest that Cav-3 V82I found in the Triton insoluble fraction does not primarily come from plasma membrane caveolae/lipid rafts but instead from intracellular compartments such as LB where Cav-3 V82I accumulates (Figure 5). To this regard, it is interesting to note that Cav-3^DGV^ LB pool is Triton-insoluble [40].

When proteasome activity is inhibited, intracellular retained and unstable Cav-3 mutants are expressed at significantly higher level and display a characteristic redistribution to ER (but not to plasma membrane) [41]. Considering that V82I mutation increased Cav-3 cell expression and that LBs have an ER origin [39], we hypothesized that Cav-3 V82I would also display a similar ER retention. However, no significant accumulation of Cav-3 V82I protein was observed within the ER (Figure 6).

Several studies have proposed a critical role for caveolin-3 in cell life and death decision mechanisms. In fact, when caveolin-3 dependent signalling pathways are disrupted, critical cellular functions are significantly affected, including proliferation [47], survival response to stressful stimuli [22], preservation of plasma membrane integrity during mechanical stresses [24] or toxin-induced membrane injury [27]. Based on these findings, we compared the survival response to brief hyperosmotic stress elicited in cells transfected with Cav-3 WT or with Cav-3 V82I. Under resting conditions, neither WT nor V82I form had any effect on cell viability (Figure 9). However, V82I mutant significantly increased cell death susceptibility under a mannitol stress that was sub-lethal in Cav-3 WT transfected cells (Figure 9). Such increased cell vulnerability may be related to the altered caveolin-3 distribution observed in Cav-3 V82I transfected cells (Figures 4, 5). In fact, the reduced plasma membrane targeting of V82I mutant would impair caveolae formation (a process that require caveolin protein) and consequently affect caveolae-mediated protective response to plasma membrane stress [24,27].

Since ERK signalling pathways are regulated by Cav-3 proteins [48] and influence the survival rate of the cells [30,49], we also explored the effect of Cav-3 V82I on ERK activation. We found that V82I significantly reduced ERK activation both under resting condition and after hyperosmotic stress (Figure 8). The negative effects on ERK activation exerted by V82I mutant may be simply related to the higher caveolin-3 expression level observed in Cav-3 V82I transfected cells. Collectively, our data suggest that the expression of Cav-3 V82I is detrimental for the survival response to mechanical stress.

Signs of apoptosis among myocytes have been documented as characteristic histological signature in various arrhythmic patients [11,50,51] and abnormal cardiomyocyte death may be clinically relevant for the development of life-threatening arrhythmia in terms of associated (if not causative) mechanism [9-11,50,51]. It is interesting to note that for Cav-3 the so-called “TFT mutation” causes apoptosis in skeletal muscle cells [52] and decreases myotube resistance to oxidative stress [22]. In connection to these data, we propose that the Cav-3 V82I variant increases cell vulnerability to stress conditions and may participate in determining the susceptibility to cardiac death.

The sodium/calcium exchanger 1 (NCX1) is an electrogenic cardiac Ca^2+^ transporter protein that regulates cardiac excitation-contraction coupling [53]. NCX1 normally contributes to the ion currents responsible for the action potential but can trigger arrhythmias under pathological conditions [53]. Based on previous data showing Cav-3/NCX1 interaction [54], we hypothesize that Cav-3 V82I could also affect NCX1 activity. However, in biochemical and functional experiments using BHK cells stable expressing NCX1, we found no interaction between NCX1 and WT or V82I Cav-3 (Additional file 4).

## Conclusions

The biochemical and functional findings presented here provided evidence that the new putative caveolin-3 V82I variant discovered in our patient has a cell expression profile significantly different not only from the WT form (Figures 2, 3, 4, 5, 6, 7, 8, 9 and Additional files 2, 3) but also from most of the mutants analyzed so far [14,15,19,35,36,41,47]. Cells expressing Cav-3 V82I displayed altered caveolin-3 distribution and were more vulnerable to sub-lethal osmotic stress. These defects may potentially be of clinical relevance considering that abnormal loss of myocyte can deadly impair people’s health [9-11,50,51].

Some Cav-3 mutants have been reported to influence the activity of ion channels localized within caveolae [12,13,55], but it is still not fully understood whether and how these mutants impact on the action potential as a whole [56,57]. Therefore, we cannot exclude the possibility that Cav-3 V82I may alter the currents carried by these channels and further work is required to address this issue. However, we provided evidence that the V82I mutant has no effect on NCX1, a key plasma membrane transporter that under normal and pathological conditions influences multiple properties of cardiomyocytes, including cell excitability [53].

## Competing interests

The authors declare that they have no competing interests.

## Authors’ contributions

VL, AAN, FA and MP designed and performed the research; AT, SG and SA supervised the project; VL and AAN wrote the manuscript with input from other authors. All authors read and approved the final manuscript.

## Authors’ information

Adriano Tagliabracci and Salvatore Amoroso are senior authors.

Annamaria A. Nasti’s present address: Department of Molecular Medicine, University of Padua, Italy.

## Supplementary Material

Additional file 1 **Identification of the missense mutation in *Cav-3 *** **gene.** Electropherograms show partial *Cav-3* sequences from control subject (top) and the index patient (bottom). The arrows indicate the heterozygous nucleotides of G/A in the proband or the homozygous nucleotides of G/G in unrelated normal control. Click here for file

Additional file 2 **Characterization of Cav-3 WT and Cav-3 V82I expression in BHK cells. The figure shows the complete Z-stack series of Cav-3 WT (a) and Cav-3 V82I (b) transfected BHK cells reported in Figure** 2**.** ~4 μm optical slice thickness, 5 z-sections collected at 1 μm intervals. Scale bar: 10 μm. Click here for file

Additional file 3 **Characterization of Cav-3 WT and Cav-3 V82I expression in H9c2 cells.(a)** H9c2 cells were transiently transfected with either Cav-3 WT or Cav-3 V82I. Twenty-four hours after transfection, cells were lysed and subjected to western blot analysis using anti-caveolin-3 antibody. The mutant form was expressed at significantly higher level than achieved with the wild-type caveolin-3. The blots are representative of 4 separate experiments. **(b)** In Cav-3 V82I transfected cells, caveolin-3 protein was located mainly in ring-shaped LB as revealed by immunofluorescence analysis. Scale bar: 5 μm. Data are representative of 2 independent transfected cultures. Click here for file

Additional file 4 **Analysis of Cav-3 WT and Cav-3 V82I interaction with NCX1 in BHK cells.(a)** Stable NCX1 expressing BHK cells were transiently transfected with EGFP, Cav-3 WT or Cav-3 V82I. Twenty-four hours after transfection, cells were lysed and subjected to western blot analysis using anti-NCX1 antibody. No difference in NCX1 expression was seen between the three transfected groups (the blot is representative of 3 separate experiments). **(b)** NCX1 was not associated with Cav-3 WT or Cav-3 V82I as determined by co-immunoprecipitation (the blots are representative of 2 separate experiments). **(c)** Representative time course of [Ca^2+^]_i_ in Fluo-4 AM loaded cells. NCX1 activity was probed in reverse mode by monitoring the intracellular Ca^2+^ increase in response to a stepwise reduction of external Na^+^ (140 mM, iso-osmotically replaced by lithium). Fluorescence is reported as ratios (F_i_/F_0_) of fluorescence counts (F_i_) relative to averaged baseline values before Na^+^ removal (F_0_). **(d)** Ca^2+^ responses (expressed as Δ%) were not significantly different between Cav-3 WT and Cav-3 V82I transfected BHK-NCX1 cells (134.0 ± 6.4% vs 138.9 ± 8.7%, WT vs V82I respectively; P = 0.7). Each bar represents the mean ± SEM of > 157 cells recorded in 3 different sessions. Click here for file
