# Supplementary figures and images for: Identification and functional analysis of a new putative caveolin-3 variant found in a patient with sudden unexplained death
Source: J Biomed Sci. 2014 Jun 10;21(1):58. doi: 10.1186/1423-0127-21-58 (PMC4109384; doi:10.1186/1423-0127-21-58)

WT

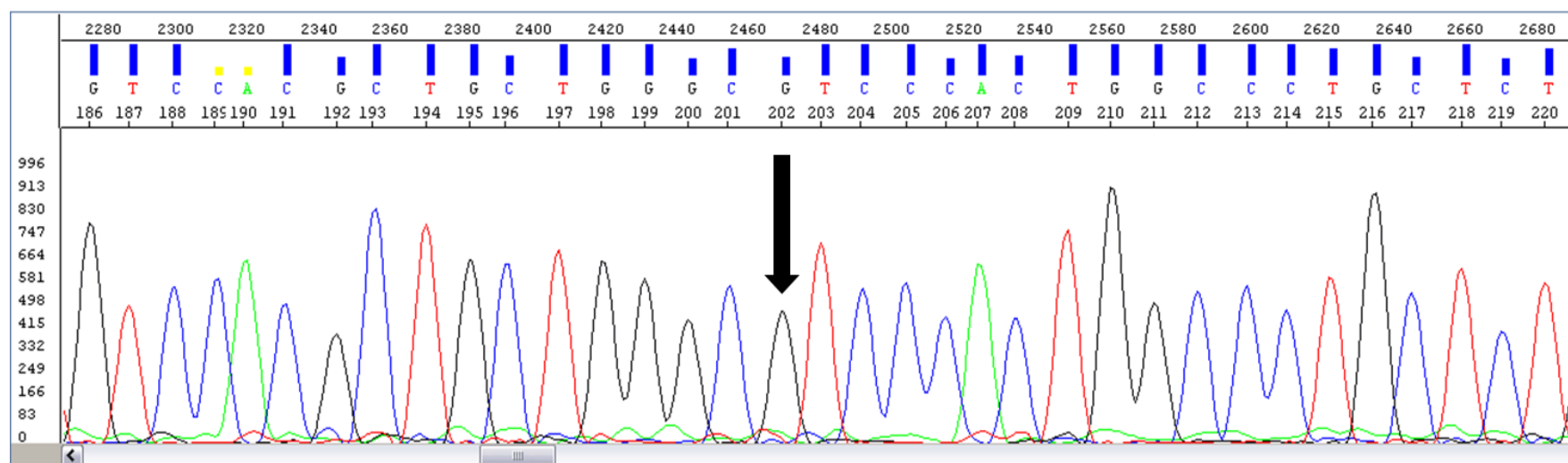

V82I

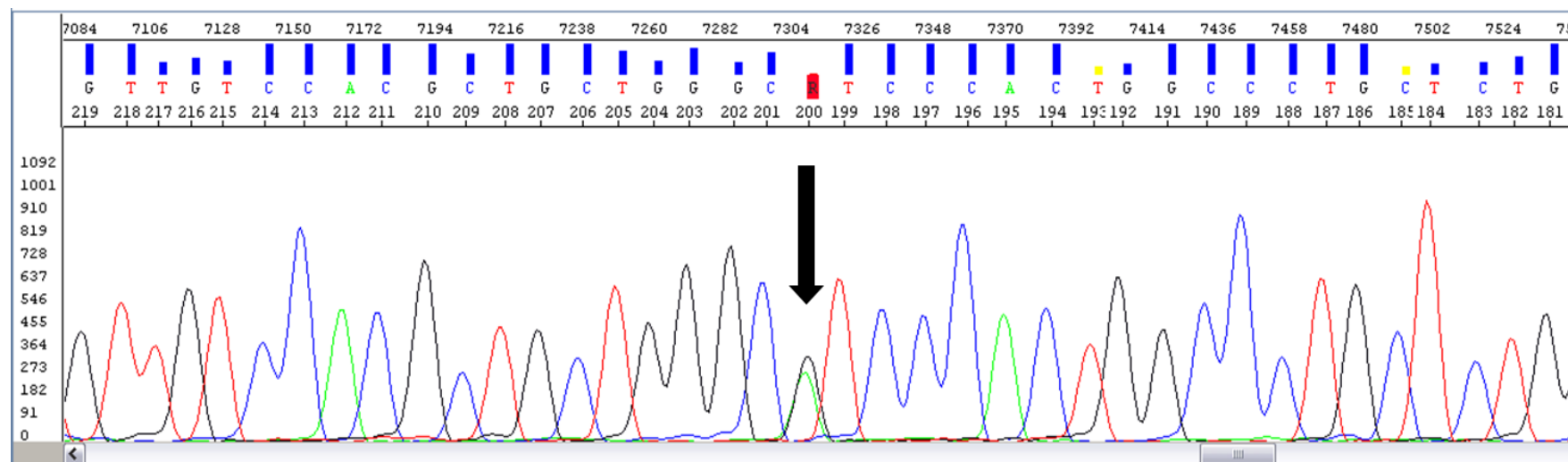

Fig. S1

Supplement: Additional file 1 — Identification of the missense mutation in Cav-3 gene. Electropherograms show partial Cav-3 sequences from control subject (top) and the index patient (bottom). The arrows indicate the heterozygous nucleotides of G/A in the proband or the homozygous nucleotides of G/G in unrelated normal control. [file 1423-0127-21-58-S1.pdf]

**a**

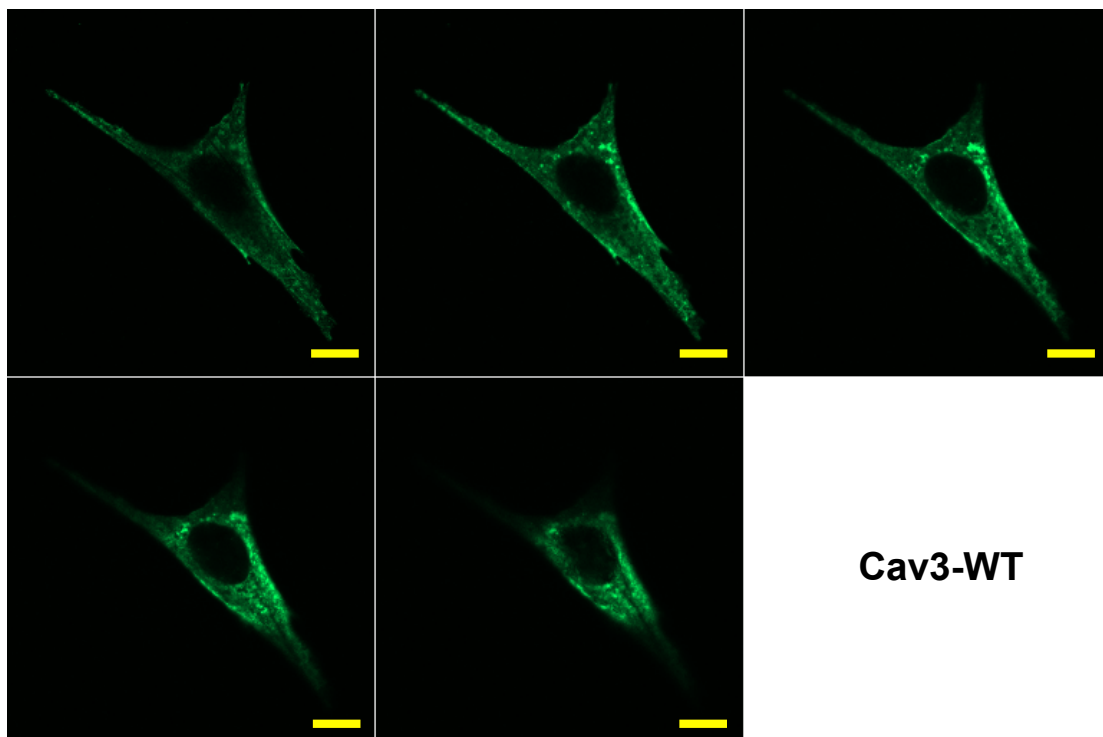

**b**

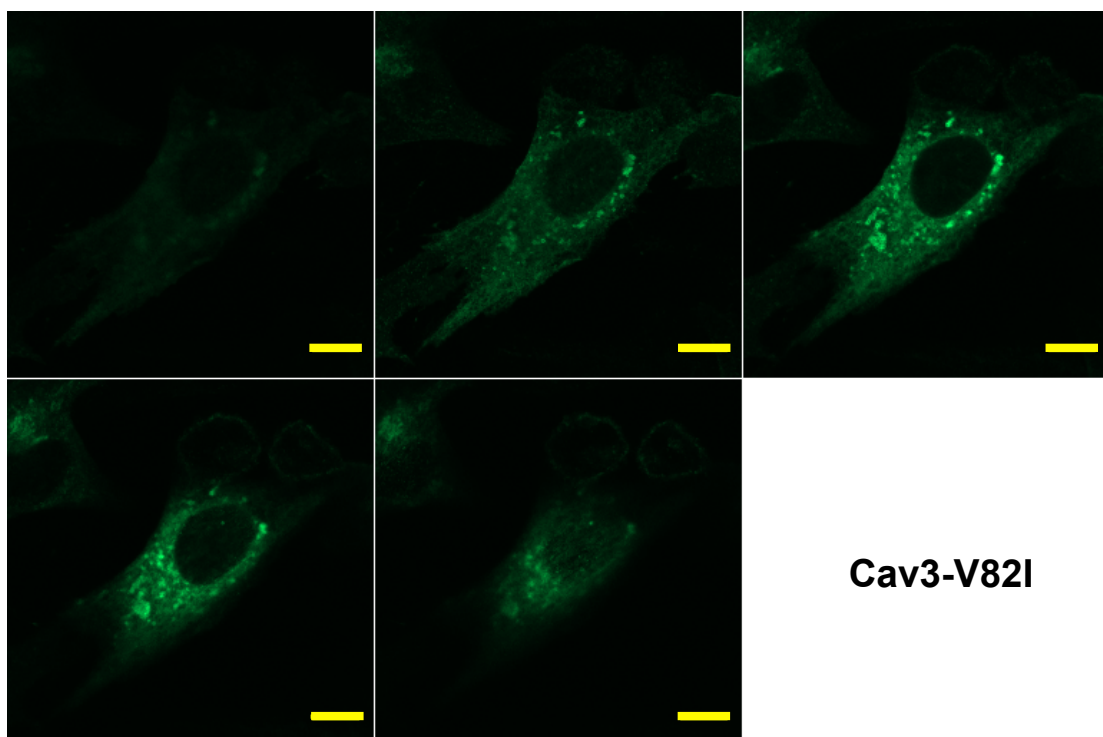

**Fig. S2**

Supplement: Additional file 2 — Characterization of Cav-3 WT and Cav-3 V82I expression in BHK cells. The figure shows the complete Z-stack series of Cav-3 WT (a) and Cav-3 V82I (b) transfected BHK cells reported in Figure 2. ~4 μm optical slice thickness, 5 z-sections collected at 1 μm intervals. Scale bar: 10 μm. [file 1423-0127-21-58-S2.pdf]

**a**

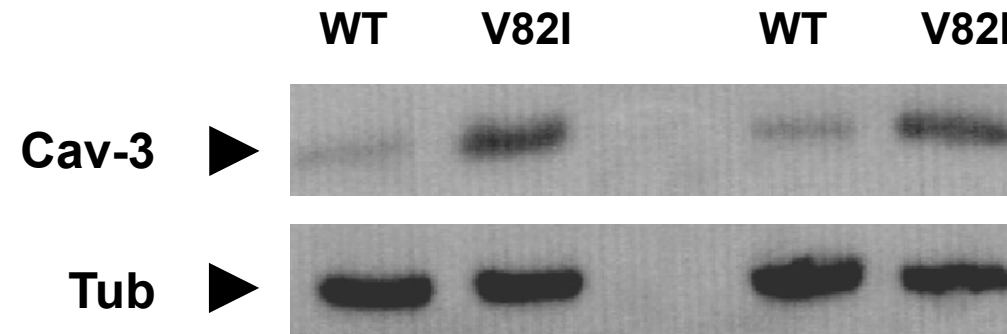

**b**

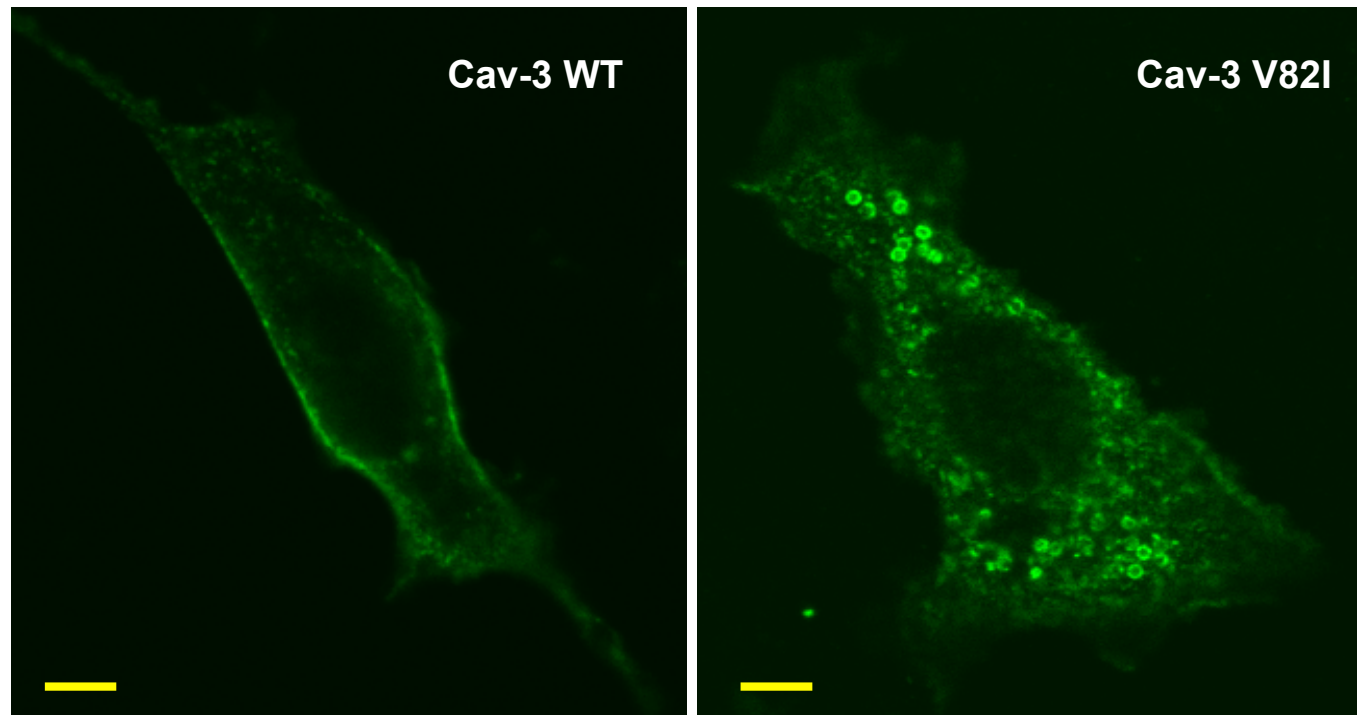

**Fig. S3**

Supplement: Additional file 3 — Characterization of Cav-3 WT and Cav-3 V82I expression in H9c2 cells.(a) H9c2 cells were transiently transfected with either Cav-3 WT or Cav-3 V82I. Twenty-four hours after transfection, cells were lysed and subjected to western blot analysis using anti-caveolin-3 antibody. The mutant form was expressed at significantly higher level than achieved with the wild-type caveolin-3. The blots are representative of 4 separate experiments. (b) In Cav-3 V82I transfected cells, caveolin-3 protein was located mainly in ring-shaped LB as revealed by immunofluorescence analysis. Scale bar: 5 μm. Data are representative of 2 independent transfected cultures. [file 1423-0127-21-58-S3.pdf]

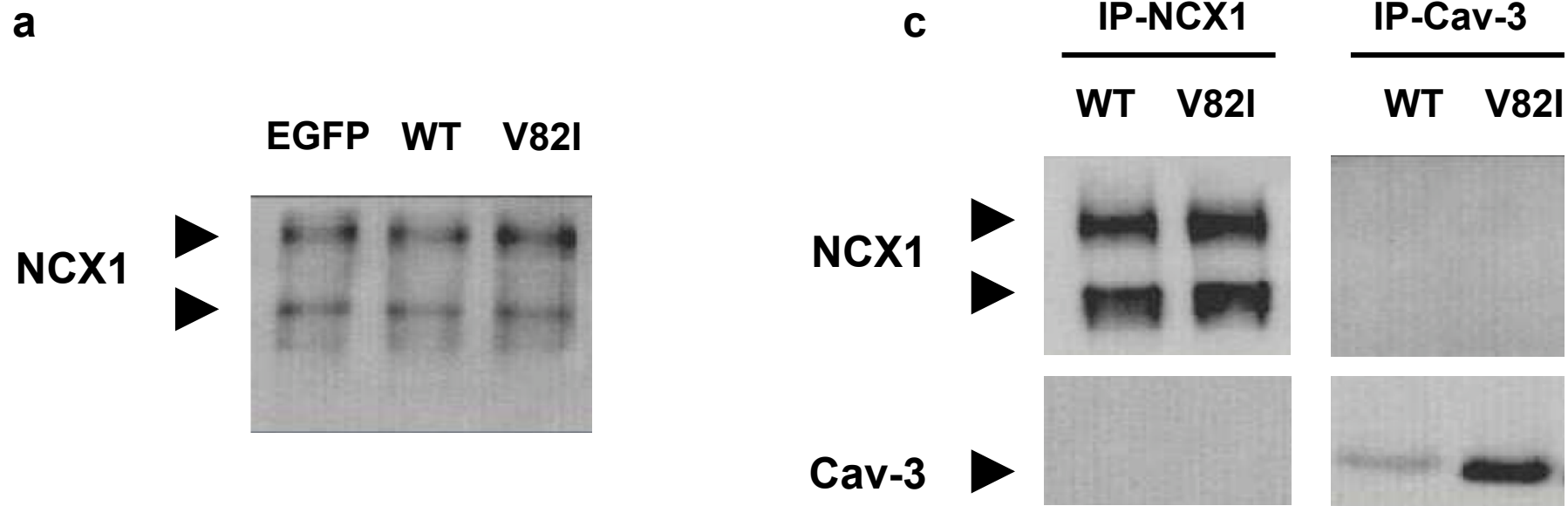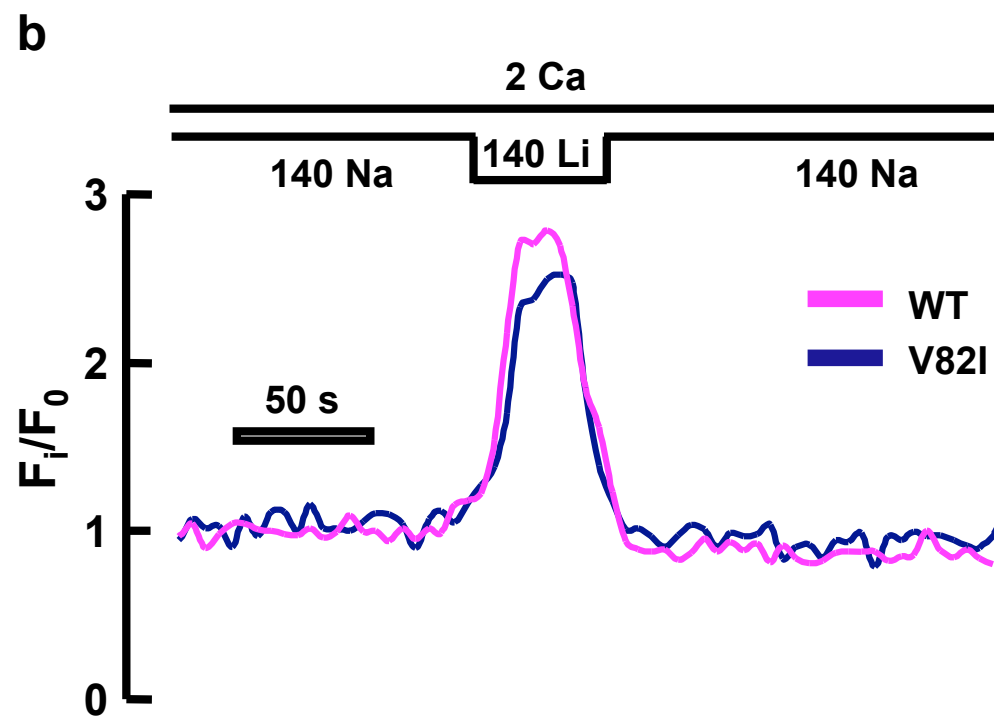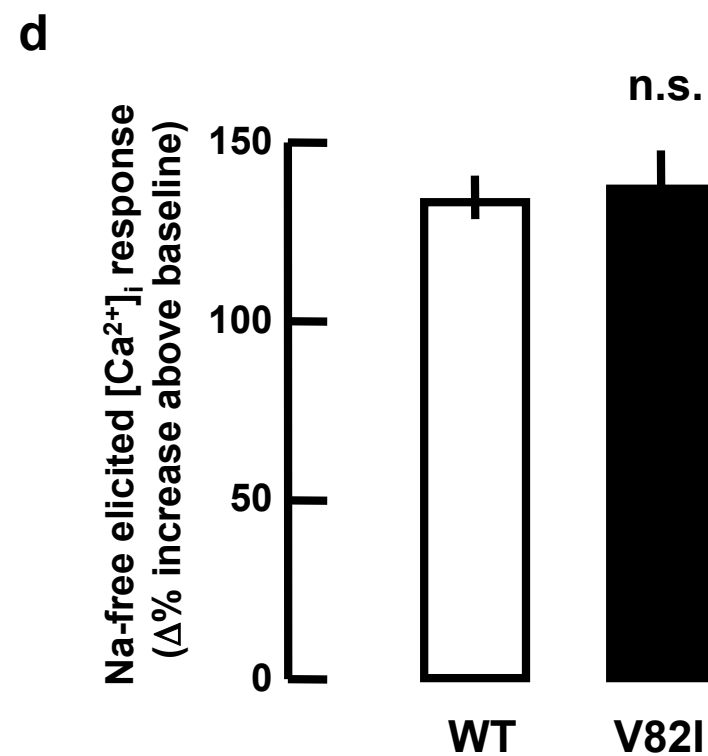

Fig. S4

Supplement: Additional file 4 — Analysis of Cav-3 WT and Cav-3 V82I interaction with NCX1 in BHK cells.(a) Stable NCX1 expressing BHK cells were transiently transfected with EGFP, Cav-3 WT or Cav-3 V82I. Twenty-four hours after transfection, cells were lysed and subjected to western blot analysis using anti-NCX1 antibody. No difference in NCX1 expression was seen between the three transfected groups (the blot is representative of 3 separate experiments). (b) NCX1 was not associated with Cav-3 WT or Cav-3 V82I as determined by co-immunoprecipitation (the blots are representative of 2 separate experiments). (c) Representative time course of [Ca2+]i in Fluo-4 AM loaded cells. NCX1 activity was probed in reverse mode by monitoring the intracellular Ca2+ increase in response to a stepwise reduction of external Na+ (140 mM, iso-osmotically replaced by lithium). Fluorescence is reported as ratios (Fi/F0) of fluorescence counts (Fi) relative to averaged baseline values before Na+ removal (F0). (d) Ca2+ responses (expressed as Δ%) were not significantly different between Cav-3 WT and Cav-3 V82I transfected BHK-NCX1 cells (134.0 ± 6.4% vs 138.9 ± 8.7%, WT vs V82I respectively; P = 0.7). Each bar represents the mean ± SEM of > 157 cells recorded in 3 different sessions. [file 1423-0127-21-58-S4.pdf]
